# Protection of coral reef fish delivers ecosystem-critical biocontrol of coral-eating starfish across the Great Barrier Reef

**DOI:** 10.1038/s41559-025-02916-z

**Published:** 2025-11-28

**Authors:** Scott A. Condie, Diego R. Barneche, Leanne M. Currey-Randall, Frederieke J. Kroon, Javier Porobic, Daniela M. Ceccarelli

**Affiliations:** 1 CSIRO Environment, Hobart, Tasmania Australia; 2 Centre for Marine Socioecology, Hobart, Tasmania Australia; 3 Australian Institute of Marine Science, Crawley, Western Australia Australia; 4 Oceans Institute, The University of Western Australia, Crawley, Western Australia Australia; 5 Australian Institute of Marine Science, Townsville, Queensland Australia

**Keywords:** Ecological modelling, Invasive species, Population dynamics, Conservation biology, Ecological networks

## Abstract

While biological control (or biocontrol) is an established method for managing pest species in terrestrial systems, few successful applications have been reported for marine environments. Crown-of-thorns starfish (CoTS, *Acanthaster* ssp.) are regarded as a pest species across the Indo-Pacific, where they are voracious predators of corals and represent one of the largest causes of coral mortality on the Great Barrier Reef (GBR). The role of reef fish in moderating outbreaks of CoTS through biocontrol has recently become more widely recognized. Here we have incorporated reef fish into a meta-community model of the GBR to demonstrate the critical role that marine reserves and other fisheries regulations have had in limiting the prevalence of CoTS outbreaks and maintaining the resilience of the GBR ecosystem. Our results suggest that without these interventions, the GBR would have already passed a major tipping point to a new state characterized by few predatory fish, continuous CoTS outbreaks and substantially lower coral cover. Model projections to 2050 demonstrate the importance of maintaining protection into the future and suggest that additional gains can be made over the next decade by continuing to manually control CoTS numbers. However, beyond 2040, the escalating impacts of climate change and the underlying resilience of CoTS populations will limit the effectiveness of interventions based on biocontrol.

## Abstract (web summary)

Incorporating reef fish into a meta-community model of the Great Barrier Reef suggests that fish-protecting interventions, including marine protected areas, have prevented the reef from already passing a tipping-point transition to a state of continual outbreaks of crown-of-thorns starfish and reduced coral cover.

## Main

Invasive pests are a major threat to global biodiversity^1^ and the functioning of ecosystems^2^, and they have large economic costs^3^. While biological control (or biocontrol) is often viewed as the most practical option for managing pests across large geographical areas, there are substantial risks associated with the introduction of novel organisms^4^. Conservation biocontrol provides much lower risks by protecting or enhancing populations of native species that are also the natural predators of pest species^5^. While traditionally applied in agricultural settings^6^, conservation biocontrol has also been proposed as a potential solution to a number of invasive marine species^7–9^. It is also likely that traditional conservation strategies such as marine reserves have been contributing to the control of pest species by protecting natural predators, even where biocontrol was not their original purpose.

We consider how marine reserve zoning and other fisheries regulations on the Great Barrier Reef (GBR) contribute to biocontrol of crown-of-thorns starfish (CoTS; *Acanthaster* spp.). CoTS are voracious coral predators, with population outbreaks periodically devastating coral reef ecosystems throughout the Indo-Pacific^10–12^. Multiple outbreaks of CoTS have occurred on the GBR over the past four decades, each beginning in the north and propagating southwards over a period of 10–15 years^13^. While the drivers of these outbreaks are still under debate^11^, their impacts, particularly when compounded by the destructive effects of escalating climate change, are severe^14^.

CoTS are arguably the only agent of large-scale coral loss that lends itself to direct local management action, with potential for immediate and tangible benefits in the form of mitigation of coral loss^15–17^. CoTS management on the GBR has evolved from manual removal of starfish at individual reef sites in the 1980s to intensive culling at high-value tourism sites to the current multi-million-dollar CoTS control programme with multiple vessels deployed to more than 200 prioritized reefs across the GBR every year^17^. Recent control efforts have been supported by extensive reef monitoring programmes able to track the progress of outbreaks^18^, as well as the development of reef meta-community models capturing outbreak dynamics and starting to provide predictive capabilities^19–22^.

Other innovative options are also being explored to control CoTS densities. A number of these options are based on the predator-removal hypothesis, which postulates that an important cause of CoTS population outbreaks is a decline in the abundance of their predators, which at natural population densities would effectively regulate CoTS abundance and prevent extensive coral losses^23^. This hypothesis implies that existing measures to protect predatory reef fish, such as no-take marine reserves and other fisheries regulations, may already be making an important unmeasured contribution to CoTS control and that increased protection may further enhance the effectiveness of biocontrol.

More than 100 species of fauna are known to prey on CoTS during one or more life stages^24^. While only a few fish species are capable of killing and consuming adult CoTS, a larger number of invertivores can prey on juvenile CoTS^25,26^. The list of fish species that either prey directly on CoTS or influence them indirectly through the food web include several emperors (*Lethrinus* spp.), tropical snappers (*Lutjanus* spp.) and groupers (Serranidae) that are targeted by commercial, recreational and indigenous coral reef fisheries on the GBR^27,28^. Catches of these species within the GBR Marine Park (GBRMP) have been regulated through zoning and fisheries management strategies, including protected zones where fishing is not permitted, catch and size limits, seasonal closures and gear restrictions^28,29^. CoTS densities and outbreak frequencies have since been found to be consistently lower in GBR protected zones^23,30^. This empirical link between historical overfishing and increased CoTS outbreaks supports the central proposition of the predator-removal hypothesis^24,30,31^ and motivates the current study.

While it has been confirmed that removal of predatory fish (emperors and groupers) from reefs is correlated with increased densities of CoTS^23^, the benefits of specific fish enhancement strategies are difficult to quantify on the basis of available empirical data derived from monitoring. Systems modelling offers a non-invasive approach to exploring the efficacy of various biocontrol measures and understanding how coral cover responds^32^. This step is critical given that large-scale interventions are likely to be costly and controversial, with highly uncertain outcomes^22,33,34^.

**Fig. 1:**
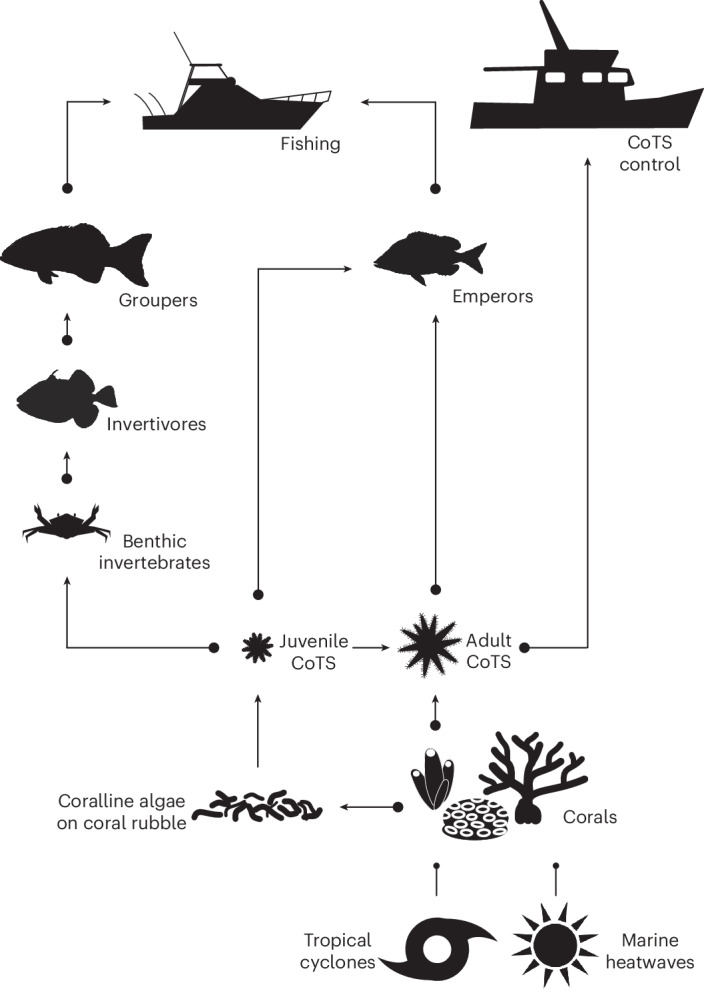
Schematic representation of the food web structure within the CoCoNet model, plus influences from environmental extremes (tropical cyclones and marine heatwaves) and human interventions (fisheries and CoTS control). The arrows indicate a positive effect and the circles indicate a negative effect. Corals were represented by five functional groups, and populations of CoTS, groupers and emperors were age structured. This ecosystem structure was replicated on 64,944 sites distributed across 3,806 reefs, with reef populations linked through marine larval dispersal estimated from modelled ocean currents. Management interventions involved modifications to fishing (zoning, other catch restrictions and stock augmentation) and CoTS control (number of control vessels and targeting on the basis of conservation zoning). This minimum realistic model only includes key interactions that are potentially capable of influencing the overall trajectory of the system. For example, trophic links between emperors and benthic invertebrates (that consume juvenile CoTS) and between invertivores and CoTS were assumed to be small relative to their other interactions. Further details on the model are provided in the Methods.

This study uses an established reef meta-community model (Fig. 1) to evaluate the efficacy of management strategies designed to enhance the levels of CoTS biocontrol on the GBR. These strategies include historical implementation of reef zoning and other fisheries management, as well as hypothetical interventions, such as expanded conservation zoning, catch restrictions, fish stock augmentation and better coordination of manual control with biocontrol. These management measures are explicitly considered for their potential value in the biocontrol of CoTS to benefit corals.

## Results

**Fig. 2:**
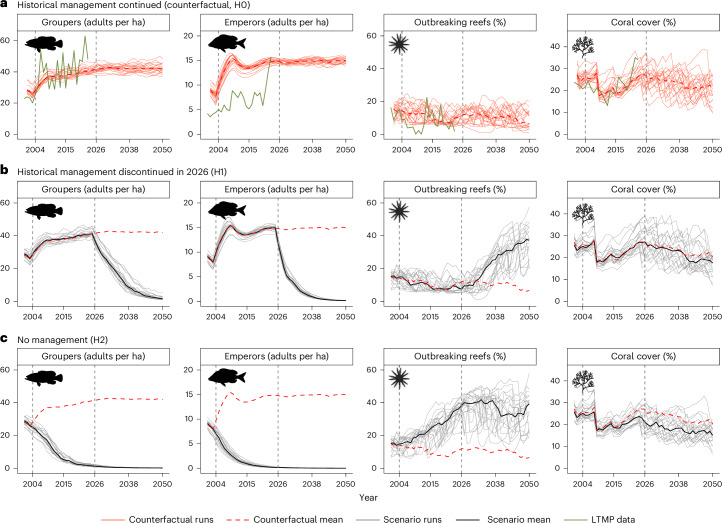
Model trends through time (2001–2050) of GBR-wide mean values under counterfactual and reduced-management scenarios. **a**–**c**, Variables include the number of mature groupers per hectare (far left), number of mature emperors per hectare (centre left), percentage of reefs with CoTS outbreaks (centre right) and percentage coral cover (far right). Scenarios correspond to the counterfactual, assuming continuation of historical management for 2026–2050 (H0) (**a**); discontinuation of all management restrictions for 2026–2050 (H1) (**b**); and hypothetical removal of all historical and future management restrictions (H2) (**c**) (Extended Data Table 2). Green lines correspond to GBR-wide means of the LTMP data. Red lines correspond to the counterfactual run (H0) with the 20 individual runs represented by thin solid lines and ensemble means by dashed lines. Thin grey lines correspond to the 20 individual runs from the scenarios and solid black lines to the ensemble means of the scenarios (H1 and H2).

Counterfactual model runs based on historical environmental conditions and historical development of management regulations (H0) produced GBR-wide trends broadly consistent with observations of groupers, emperors, outbreaking reefs and coral cover from the Long-Term Monitoring Program (LTMP) (Fig. 2a and Supplementary Figs. 1a,b). Comparing only mature fish from the model aligned relatively well with observed grouper densities, allowing for some overestimation of emperor densities to account for their lower visual detectability^35^. Observations and model results both reveal fish densities recovering following the major re-zoning of reefs and additional fisheries regulations implemented in 2004. While mean fish densities approximately doubled over the following 20-year period, the impacts on CoTS outbreaks and coral cover were more difficult to disentangle from the cumulative pressures of ongoing cyclone and heatwave events. CoTS outbreaks in the model tended to be more persistent through time than observed by the LTMP, and the recent recovery of coral evident in LTMP records was only replicated in a relatively small proportion of the model runs (Fig. 2a). However, in most respects, model responses were broadly consistent with observations at the GBR scale, confirming that the counterfactual model represented an appropriate baseline for evaluating alternative management scenarios.

**Fig. 3:**
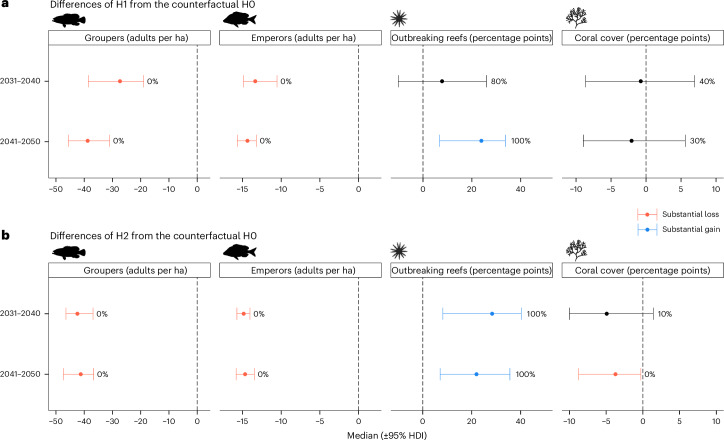
Difference from the counterfactual (H0) of scenarios with reduced levels of management. **a**,**b**, Scenarios with discontinuation of all management for 2026–2050 (H1) (**a**) and hypothetical removal of all historical and future management (H2) (**b**). Results are shown for GBR-wide mean values from 2031 to 2040 (top) and 2041 to 2050 (bottom). The median (circle) and the 95% highest density interval (HDI) across ensemble-level differences are presented (20-run ensembles), as well as the percentage of runs in which the difference was above 0. Red colour indicates substantial losses relative to the counterfactual (95% HDI fully below 0) and blue indicates substantial gains relative to the counterfactual (95% HDI fully above 0). Both scenarios show substantial reductions in fish densities; substantial increases in CoTS outbreaks (apart from H1 in the 2030s); and, for H2, substantial reductions in coral cover during the 2040s.

Discontinuing all protection of fish from 2026 (H1) triggered an ecological tipping point from which groupers and emperors declined rapidly, largely disappearing from the system by 2050 (Fig. 2b). CoTS responded with a fourfold increase in the ensemble mean percentage of reefs outbreaking by 2050. Under increasing predation pressure from CoTS, mean coral cover also declined, although not substantially within the context of variability across the ensemble (Figs. 2b and 3a). The hypothetical scenario in which no management was deployed either historically or in the future (H2) led to a much earlier decline in groupers and emperors (by 2020) and increased the size and frequency of CoTS outbreaks (Figs. 2c and 3b and Supplementary Fig. 1b,c). While the percentage of outbreak reefs reached similar peak values in scenarios H1 and H2, the decline in coral cover was more substantial in H2 after enduring 20 additional years of more frequent outbreaks (Fig. 2c and 3b). These results were relatively insensitive to predation rates of groupers and emperors, with a 50% reduction shifting coral cover differences in Fig. 3b by less than 8% (2031–2050 means from −0.0466 to −0.0432).

**Fig. 4:**
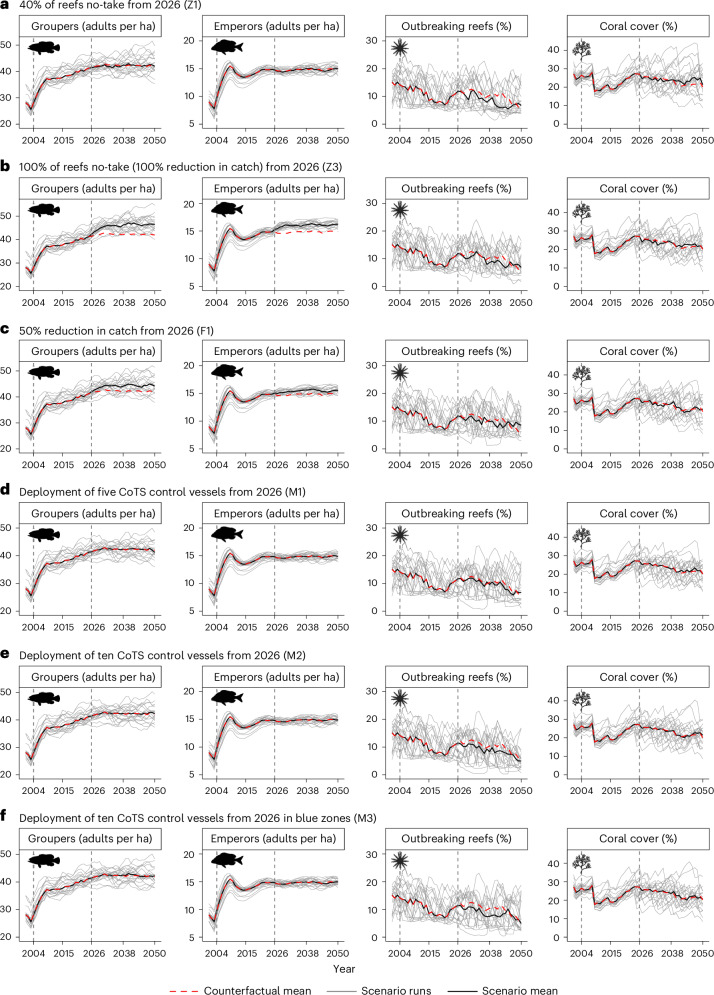
Model trends through time (2001–2050) of GBR-wide mean values under the counterfactual and increased-management scenarios. **a**–**f**, Variables include the number of mature groupers per hectare (far left), mean number of mature emperors per hectare (centre left), mean percentage of reefs with CoTS outbreaks (centre right) and mean percentage coral cover (far right). Each row corresponds to one scenario: Z1 (**a**), Z3 (**b**), F1 (**c**), M1 (**d**), M2 (**e**) and M3 (**f**) (Extended Data Table 2). Dashed red lines correspond to the ensemble mean of the counterfactual (H0), thin grey lines to individual runs from each scenario and black lines to the ensemble means from each scenario (20-run ensembles).

**Fig. 5:**
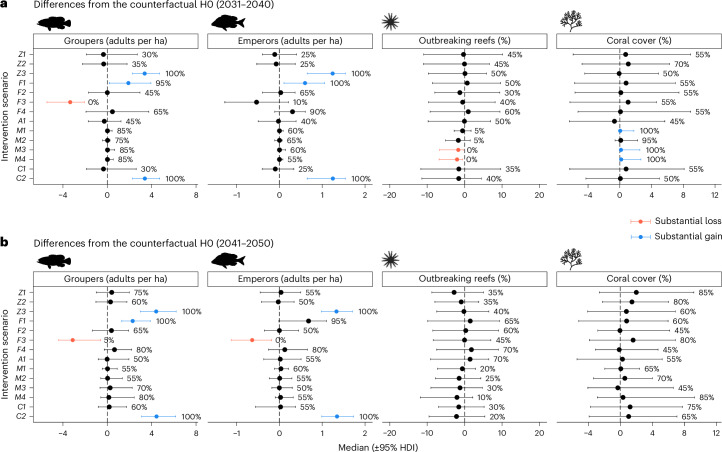
Difference from the counterfactual (H0) of scenarios with increased levels of management. **a**,**b**, Results are shown for GBR-wide mean values from 2031 to 2040 (**a**) and from 2041 to 2050 (**b**). The median (circle) and the 95% HDI across ensemble-level differences are shown (20-run ensembles), as well as the percentage of runs in which the difference was above 0. Red colour indicates substantial losses relative to the counterfactual (95% HDI fully below 0), and blue indicates substantial gains relative to the counterfactual (95% HDI fully above 0). All management scenarios are defined in Extended Data Table 2.

Increasing the fraction of protected reefs from 30% to 40% (scenario Z1, Fig. 4a) or 60% (scenario Z2) in 2026, without reducing GBR-wide fisheries catch, had little effect on grouper or emperor densities. While the percentage of reefs outbreaking decreased and mean coral cover increased, the effects were not substantial (Fig. 5). When catch was reduced by 50% (scenario F1, Fig. 4c) or 100% (scenario Z3, Fig. 4b), there was a substantial increase in grouper and emperor abundances, although this again did not result in substantial changes in CoTS outbreaks or coral cover (Fig. 5). Imposing size limits on catches (scenarios F3 and F4) or boosting emperor stocks through releases of juvenile fish (scenario A1) were also ineffective, with upper size limits (scenario F3) unexpectedly reducing fish densities substantially (Fig. 5). Overall, the zoning and fisheries management scenarios indicate that regulations that displace fishing, rather than reduce fishing, tend to negatively impact fish stocks at the GBR-wide scale, although not at levels that substantially influence CoTS or corals.

During the 2030s, the model indicated that manual control was the most reliable strategy for reducing the number of reefs experiencing CoTS outbreaks and increasing coral cover, without affecting fish stocks (Fig. 4d–f and 5a). There were no substantial differences associated with focusing control efforts within unprotected zones (M3) or protected zones (M4). While benefits increased when the number of vessels was increased from five to ten, the rise in coral cover was modest in most model runs (Fig. 5a). During the 2040s, there was less coral and less CoTS, such that climate-driven variability tended to dominate the signal and overwhelm any intervention benefits. However, for many of the interventions (Z1, Z2, F3 and M4), there was at least an 80% probability of increased coral cover (Fig. 5b).

## Discussion

In this study, we demonstrate the critical role that zoning and fisheries management strategies implemented in the GBRMP since 2004 (ref. ^36^) have had in recovering predatory fish populations, reducing CoTS outbreaks and mitigating coral loss. Model scenarios suggest that without these interventions, grouper and emperor populations on the GBR would have consistently declined under increasing fishing pressure and potentially become functionally extinct by 2025. The simulated decline in natural predators also led to a new ecological state^37^ with much higher rates of CoTS outbreaks (40% versus 12% ensemble mean by 2030) and lower coral cover (20% versus 26% ensemble mean by 2030), together indicating a loss of resilience. Similar regime changes resulted from simulated removal of zoning and fisheries regulations in 2026. These results suggest that the 2004 management reforms potentially averted a major tipping point on the GBR involving predator loss (fish and invertebrates), subsequent meso-predator release (CoTS) and then the decline of the key ecosystem engineer in the system (corals)^38–40^. Analogous transitions in terrestrial systems, often involving removal of predators that graze on habitat-forming plants, have been characterized as ecological meltdowns^41,42^.

While historical management interventions delivered major benefits by averting a detrimental regime change, additional zoning or fisheries controls provided less certain returns in terms of both CoTS outbreak reductions and mitigation of coral losses. While expanding protected zones or reducing catches enhanced stocks of groupers and emperors, gains in coral cover tended to be overwhelmed by external drivers such as climate-related mortality^19,33^. The strategies also had their own inherent limitations. For example, declaring additional protected zones without reducing overall catches displaced fishing effort into the remaining unprotected zones^43,44^, potentially leaving them highly exposed to CoTS outbreaks. Equally, suppressing CoTS outbreaks by enhancing either biocontrol or human manual control protected more coral communities, but such a change may also have prevented the complete collapse of CoTS populations (owing to starvation) and allowed them to persist at lower densities. While this substitution of acute outbreaks with more chronic outbreaks may help to stabilize both CoTS and coral populations^45^, the overall effect could be to slow coral recovery.

These findings have important implications for the ongoing management of the GBR and other reef systems. Clearly, the threat of ecosystem regime change vindicates the zoning and other fisheries regulations implemented through to 2004^46^. From this perspective, the model results provide a strong evidence base to defend the existing management arrangement should the need arise. Equally important is the finding that further expansion of these approaches will probably provide diminishing positive returns, while also potentially triggering opposition amongst local communities^43,47^, as support for strong climate action continues to vacillate within the Australian population^48^. From a reef-management perspective, these trade-offs may become even less attractive as more novel interventions to protect coral reefs become available^19,49,50^. Direct manual control of CoTS is less controversial and likely to provide more reliable outcomes in reducing CoTS outbreaks and increasing coral cover. However, making substantial gains at the scale of the GBR will almost certainly require a expansion of the existing control fleet^17,21,22^. While a relatively diverse range of interventions have been identified (in close consultation with management agencies) and explored here, modelling of more bespoke strategies may uncover further potential. For example, current prioritizing of reefs for CoTS control on the basis of their potential as sources of coral larvae or CoTS larvae^51,52^ could be extended to take into account their status as potential climate refugia^53^.

Results from this study clearly need to be interpreted within the context of underlying uncertainties of the Coral Community Network (CoCoNet) model. The GBR is one of the most complex ecosystems on Earth, and the model only attempts to capture the most important components and processes controlling the long-term trajectory of the system. For example, while predation of adult CoTS by giant triton (*Charonia tritonis*) has been well documented, their current rarity on the GBR precludes any substantial influence on CoTS populations^54^ and hence they have been omitted from the model trophic structure (Fig. 1). While there are no technical impediments to adding additional components and processes to the model, increasing complexity can increase errors and reduce the relevance and usefulness of models^55–57^. This is particularly problematic where the spatio-temporal scales of these processes are mismatched to the model, often leading to large aggregation errors^58^. Even our parsimonious approach required assumptions that may be contested (Supplementary Table 1). Acknowledging that complex system models can never be fully verified^59^, the formulation has been validated quantitatively here and previously^19,21,60,61^ by comparing outputs with available LTMP data (positivism perspective^62^) and qualitatively in terms of its fitness for purpose through continuous evaluation by experts in large programmes such as the Reef Restoration and Adaptation Program and the CoTS Control Innovation Program (relativism perspective^62^).

Even within the context of a minimum realistic model, key ecological processes including predation on juvenile CoTS^26^, sub-lethal effects such as behavioural suppression or reduced fecundity^14^ and the collapse of CoTS outbreaks are still poorly understood^11^. The representation of fisher behaviour in the model is also challenging to quantify, particularly in relation to compliance with zoning and other regulations^63^ and the contribution of recreational fishers^27,64^. With multiple sources of irreducible uncertainty, LTMP observations provide critical points of comparison with the model hindcast that improve confidence in our projections. However, only a small proportion of GBR reefs can be monitored and the detectability of fish and CoTS associated with the monitoring programme’s protocols require calibration^35,65^.

Given high levels of both climate uncertainty^33^ and model uncertainty^22^, capturing stochastic variability through an ensemble-modelling approach was essential in representing both hindcast and projected future conditions on the GBR. These uncertainties were further mitigated by limiting our analysis and interpretation in two key aspects. First, model ensembles were identical (down to the random seeds used for stochastic processes), with our analyses focusing on changes relative to the counterfactual, rather than absolute levels that are more sensitive to systematic model biases. Second, all results were presented as GBR-wide means. This acknowledges that in a complex nonlinear predator–prey system (with unknown initial populations distributions), populations at the scale of individual reefs or small regions cannot be reliably predicted. A limitation of this focus on large-scale responses is that more localized benefits have not been explored. For example, re-zoning may provide specific benefits to reefs downstream of newly protected zones.

Acknowledging these inherent uncertainties, this study represents an important step towards understanding the potential of conservation biocontrol for protecting the GBR under the increasing threat of climate change. Systems modelling provides our only means of projecting multiple scenarios into the future and testing the efficacy of interventions at the scale of the GBR without costly and unpopular changes to real-world management. It has provided clear support for the benefits of biocontrol through the protection of fish predators and suggests that further model refinement and collection of key empirical evidence is warranted.

## Methods

### The CoCoNet model

The CoCoNet model is a meta-community model developed in the NetLogo environment (version 6.4.0) to represent key physical, ecological and human processes on the GBR. It was developed to explore the effects of physical and ecological drivers on the health of coral reef systems both historically and into the future under the influences of climate change and potential management interventions. A wide range of management interventions have previously been simulated within CoCoNet, including manual CoTS control through culling^19,21,22^. Exploring the potential benefits of biocontrol in the current study has required the inclusion of reef fish and related fisheries, informed by fisheries catch data and reef fish monitoring data. Because most aspects of the model have previously been described in detail^19,21,22,61,66^, we provide here only a summary description, emphasizing new developments implemented to meet the objectives of the current work. All model equations and parameter values are listed in the Supplementary Information.

### Reef ecology

CoCoNet represented communities of corals by five broad functional groups (Extended Data Table 1) distinguished within the model in terms of their growth rates, susceptibility to environmental impacts such as cyclones and marine heatwaves and their vulnerability to CoTS predation^67–69^. CoTS populations were size-structured, differentiating larvae (age 0 years), herbivorous juveniles (age 1 year) and five corallivorous adult classes (ages 2, 3, 4, 5 and 6+ years). Trophic interactions between corals and CoTS were calculated using a formulation that included a delay in the juvenile-to-adult transition when coral cover was low^70^; doubling of adult CoTS predation rates with each age class until onset of senescence from 6 years of age^71^; and dietary preference for faster-growing corals^72^, with population decline when these became rare.

Rate parameters such as growth, predation and natural mortalities were taken from empirical data wherever available or else fitted to data collected by the Australian Institute of Marine Science (AIMS) LTMP^60,73^. However, even where detailed site-specific data are available, there may be large uncertainties around how well those data generalize across regions and time frames. Importantly, highly uncertain parameters, such as larval supply scaling (described below), were always used in the context of inherently stochastic processes whereby their uncertainty could be explicitly captured within the ensemble model results.

Four additional ecological groups have been added to fulfil the objectives of the current study: (1) benthic invertebrates, such as the red decorator crab *Schizophrys aspera*^26^ that prey on juvenile CoTS; (2) invertivorous fish, such as triggerfish *Balistidae* spp.^74^ that prey on benthic invertebrates; (3) emperors, such as redthroat and spangled emperors *Lethrinus* spp.^75,76^ that prey on both juvenile and adult CoTS; and (4) groupers, such as coral trout *Plectropomus* spp.^77^ that prey on invertivorous fish (Fig. 1). Ecological processes of growth, mortality and reproduction were included for all groups, with age-structured populations tracked for CoTS, emperors and groupers (Extended Data Table 1).

### Reef connectivity

Populations of all groups were represented on 64,944 sites distributed across a network of 3,806 reefs. Each site encompassed approximately 10 ha of coral habitat, equating to the coverage of individual dives undertaken by the CoTS control programme (described below). Reef populations were connected through larval dispersal processes. Larval production was assumed to be proportional to adult population densities, with rates increasing by a factor of 4 for each CoTS age class^78,79^ and by a factor of 2 for each emperor and grouper age class^11,80,81^.

Dispersal following spawning at each reef was modelled using particle tracking techniques based on the OceanParcels code (https://oceanparcels.org) and 8 years (2015–2022) of simulated ocean currents from the eReefs 1-km resolution hydrodynamic model (GBR1:H2.0)^82–84^. Dispersal modelling used the preferred swimming depths and mortality rate of CoTS^85,86^, coral^87,88^ and fish larvae^89^. Given that currents were only resolved at a 1-km scale, all particles passing within 1 km of a reef were counted as potentially contributing to recruitment at that reef. While dispersal modelling provided detailed spatial and temporal data on relative rates of larval recruitment, absolute rates cannot be measured or verified directly^90^. Hence, CoCoNet included an additional reef connectivity scaling factor that was adjusted when calibrating the model against LTMP data. The purpose of this factor was not to oversimplify dispersal but to scale relative recruitment to realistic absolute values while preserving the spatial heterogeneity embedded in the original high-resolution larval dispersal outputs. This is a common approach in large-scale ecosystem models where absolute larval supply is unobservable.

### Environmental forcing

Reefs were exposed to environmental forcing in the forms of tropical cyclones (physical damage of corals), flood plumes (restricted growth of corals), marine heatwaves (coral bleaching and mortality) and ocean acidification (reduced coral growth and increased susceptibility to tropical cyclones). In line with the terrestrial run-off hypothesis^91–94^, previous versions of CoCoNet^19,21,61^ assumed enhanced CoTS larval survival on the inner reef owing to higher levels of nutrients and planktonic food within flood plumes. However, the overall effect of flood plumes on CoTS continues to be debated with evidence that: (1) plumes directly impact only a small proportion of inner shelf reefs^83,95^ where CoTS tend to be less abundant^96^, (2) CoTS larval development does not require eutrophic conditions^97,98^, (3) the low salinity of plume water has a strong negative effect on CoTS larval survival^99^ and (4) CoTS outbreaks occur in other parts of the world where there are no apparent terrestrial sources of nutrient enrichment^78,100^. Given the equivocal nature of the evidence and our focus on reefs offshore of the flood plume zone (where commercial fishing and CoTS control tend to operate), any direct effects of run-off on CoTS larvae have been omitted from the current study.

Historical cyclones and heatwaves were applied from 1976 to 2024, followed by stochastic projections from 2025 to 2050. With climate projections revealing no clear trend in future frequency or intensity of cyclones in the GBR region^101^, cyclone projections in CoCoNet were based on historical distributions. Conversely, heatwaves in the GBR region increase in all climate projections^102^. Scenarios reported here all used Socioeconomic Pathways SSP1-2.6 (1.8 °C global warming by 2100), which was selected as plausible and damaging to coral reefs^71^ but not so extreme as to mask potential benefits from the modelled biocontrol and manual control strategies.

### Reef fisheries

The model included catches of both emperors (red-throated and spangled) and groupers (coral trout)^27^. Commercial and charter catches recorded from 1989 to 2021 were used to estimate the probability distributions for emperor and grouper catches on the basis of offshore distance, latitude and year. These probability distributions were approximated using analytical functions in the model that extrapolated catches backwards to 1940 (assuming up to 50% unreported over this largely unregulated period) and projected forward with a fixed mean catch from 2022. Catch rates, expressed as a proportion of local abundance, were set to zero for fish under 3 years of age and uniform for all fish aged 3 years or older.

Several fisheries management changes influenced catch rates over the historical period, including a shift around 1996 towards supplying the live reef finfish trade focused on coral trout; stricter fish size limits introduced for coral trout in 1996 and redthroat emperor in 2003; and major regulatory changes in 2003–2004 that included modification of commercial licences, restructuring of the commercial line fishing fleet through a buy-back programme, introduction of individual transferable quotas, revised reef fish possession limits, gear restrictions and seasonal (spawning) fishing closures^29^. GBRMP zoning regulations were applied from 1987 when approximately 150 reefs were closed to fishing, with more than 1,000 reefs declared as no-take protected zones as part of the 2004 re-zoning^46,36^. The modelling assumed full compliance with zoning regulations, with all fishing (reported and unreported) occurring on reefs open to fishing and within legal size limits. While compliance rates are notoriously difficult to measure^63^, available estimates suggest that they are relatively high for the GBR (less than one fish per hectare per annum on average taken from protected reefs even quite close to major population centres)^103^.

### CoTS control

A CoTS control programme was first established on the GBR in 2012 and since 2018 has followed an Integrated Pest Management approach, whereby empirical and modelled data are used within a structured decision-making process to guide control efforts^16,17,104^. A key element of this approach is the prioritization of reefs for treatment on the basis of factors such as economic value (for example, tourism sites), ecological significance (for example, critical larval source reefs), vulnerability to CoTS (based on historical observations) and various logistical factors^17^. Each year, following consultation with key stakeholders, a subset of priority reefs is selected as target reefs for culling. Target reefs undergo intensive culling until an ecological threshold is reached and are then maintained with periodic surveillance. The model replicated this process annually (using the 2023 priority list generated by the CoTS Control Program) until the annual control capacity, based on the number and diver capacity of control vessels, had been fully utilized.

### Model calibration

The CoCoNet model has been calibrated against reef survey data from the AIMS LTMP. These data included estimated coral cover percentages and CoTS outbreak status along reef circumferences^105^, along with fish abundances from underwater visual surveys along fixed transects repeated yearly^18^. Calibration has been conducted at both individual-reef scale^73^ and reef-network scale^19,61^, successfully reproducing historical trajectories of regional coral cover and CoTS outbreaks, as well as emergent system responses such as coral recovery at close to its observed periodicity^61^.

The current study required some re-calibration of the model to include comparison of emperors and groupers with LTMP data. This process utilized a formal parameter estimation process followed by heuristic parameter refinement to enhance the model’s alignment with LTMP data. More traditional frequentist metrics, such as correlation and *P* values, are not informative for ensemble modelling that includes stochastic forcing elements. Specifically, there is no expectation that a particular run in the ensemble should align with historical data, nor that the ensemble mean should necessarily align with historical data. Moreover, *P* values are determined by statistical power and can therefore be made arbitrarily small by simply increasing the number of simulations^106^. Hence, more important than fitting to a single historical trajectory (frequentist perspective) is that ensemble results capture the range of plausible futures (Bayesian perspective).

### Model scenarios

Consultation with key stakeholders involved in management and support of research for the GBR (Great Barrier Reef Marine Park Authority, Queensland Department of Agriculture and Fisheries and the Great Barrier Reef Foundation) identified a series of extra biocontrol interventions that were simulated for the period 2026–2050 (Extended Data Table 2). These scenarios were designed to evaluate and compare strategies for fish protection (conservation biocontrol), fish stock enhancement (augmentative biocontrol),and manual control (human control) in terms of their efficacy in reducing the impact of CoTS predation on coral.

To capture potential variability associated with future events such as cyclones, heatwaves and CoTS outbreaks, each management scenario was run 20 times to form an ensemble of possible outcomes. Generation of each ensemble began with a 50-year spin-up (1906–1955), with the 1955 state providing the initial conditions for all 20 runs within that ensemble. Individual runs then spanned another 70 years (1956–2025), forced by historical cyclones and heatwaves, before interventions were applied for a further 25 years (2026–2050). Underlying model stochasticity ensured that each run had a distinct state when management interventions were first initiated in 2026, after which additional variability was introduced by the timing and magnitude of cyclone and bleaching events generated by the specified climate projection (SSP1-2.6).

### Reporting summary

Further information on research design is available in the Nature Portfolio Reporting Summary linked to this article.

## Supplementary information

Supplementary Information

Reporting Summary

## Figures and Tables

**Extended Data Table 1:**
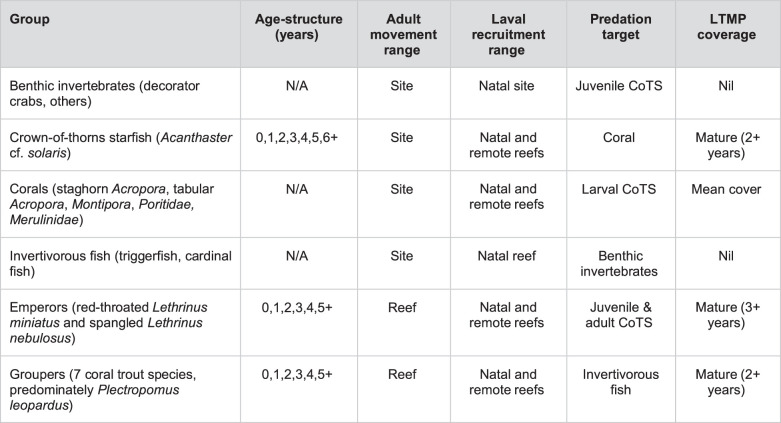
Characteristics of ecological groups included in the CoCoNet model

Key ecological characteristics of model groups and their monitoring status within the GBR Long-Term Monitoring Program (LTMP).

**Extended Data Table 2:**
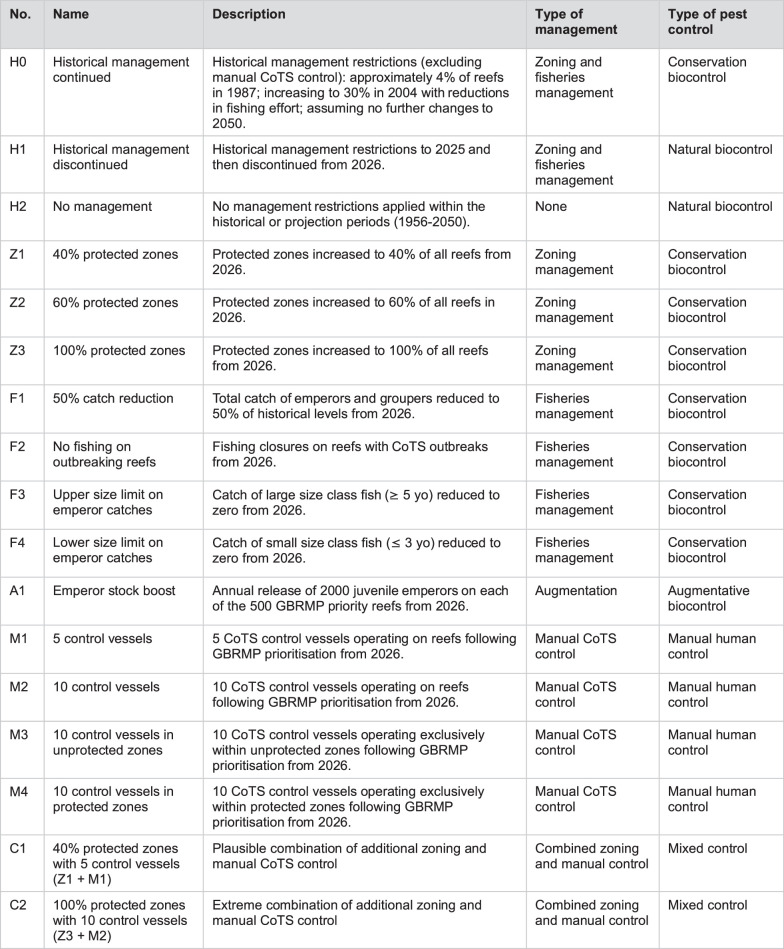
Management scenarios tested within the CoCoNet model

Continuation of historical management (scenario H0) provides the counterfactual against which all other scenarios are compared. The types of pest control have been classified within a standardized framework and do not include any instances of classical biocontrol whereby foreign organisms are introduced.

## Data Availability

The model data generated in this study can be accessed via Dryad at http://datadryad.org/share/xxGUGVuuflXrdIysXp3TJyM5BajOJYuKmItQt2AGYe8 (ref. ^107^).

## Code availability

The model code developed within this study can be accessed via Dryad at http://datadryad.org/share/xxGUGVuuflXrdIysXp3TJyM5BajOJYuKmItQt2AGYe8 (ref. ^107^).
