# Supplementary material for: Protection of coral reef fish delivers ecosystem-critical biocontrol of coral-eating starfish across the Great Barrier Reef
Source: Nat Ecol Evol. 2025 Nov 28;10(1):117–27. doi: 10.1038/s41559-025-02916-z (PMC12789020; doi:10.1038/s41559-025-02916-z)
Supplement: Supplementary file 1 — Supplementary Information [file 41559_2025_2916_MOESM1_ESM.pdf]

nature ecology & evolution

Supplementary information https://doi.org/10.1038/s41559-025-02916-z

Protection of coral reef fish delivers ecosystem-critical biocontrol of coral-eating starfish across the Great Barrier Reef

In the format provided by the authors and unedited

Supplementary information:

Protection of coral reef fish delivers ecosystem-critical biocontrol of coral-eating starfish across the Great Barrier Reef

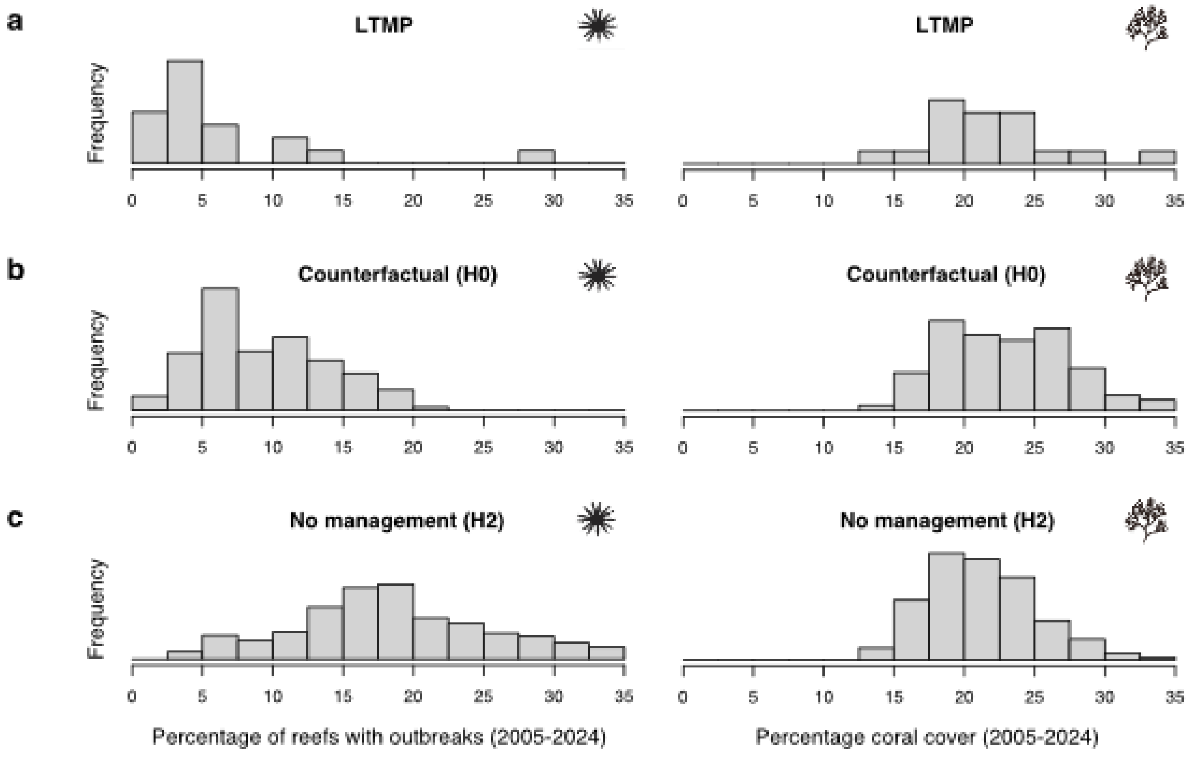

Supplementary Figure 1: Histograms of percentage of reefs with CoTS outbreaks (left) and percentage coral cover (right) for the 20-year period 2005-2024. Results are from: (a) the LTMP observations; (b) the counterfactual model run (H0); and (c) the hypothetical removal of all historical and future management restrictions run (H2). Each of the model-based histograms combined data from ensembles of n = 20 runs. There is broad consistency between observed distributions (a) and the counterfactual (b), although simulated outbreaks tend to be more persistent than observed. Simulated removal of all historical regulation (c) resulted in much more persistent outbreaks and shift in the distribution towards lower coral cover.

Supplementary Table 1: CoCoNet model equations relating to age-stuctured CoTS populations and coral functional groups. The coral groups include: *sa* = staghorn acropora; *ta* = tabular acropora; *tt* = thermally tolerant; *mo* = montipora; *po* = poritidae; and *fa* = favids.

Description	Equation	Assumptions	#
Crown-of-thorns starfish population dynamics			
CoTS: age 0	$S_{y+1,0} = \alpha^S R_y \left(\lambda(S_{y,2} + 2S_{y,3} + 4S_{y,4} + 8S_{y,5} + 8S_{y,6+}) + (1-\lambda)\sum_{reefs} \mathrm{K}_y^S(\dot{S}_{y,2} + 4\,\dot{S}_{y,3} + 16\,\dot{S}_{y,4} + 32\dot{S}_{y,5} + 32\dot{S}_{y,6+})\right)$	CoTS settlement limited to rubble area in part due to predation and stinging by corals [1]. Fecundity of CoTS doubles with each size class [2] before plateauing at 6-years with senescent phase.	1a
CoTS: age 1	$S_{y+1,1} = \left(S_{y,0} + S_{y,1}\left(1 - C_y^{f\,0.5}\right)\right)e^{-\left(M_1^S/R_y\right)} - q_{y,1}^{SB} - q_{y,1}^{SE}$	Natural mortality of juvenile CoTS dependent on availability of their preferred coral rubble habitat.	1b
CoTS: age 2	$S_{y+1,2} = S_{y,1}C_y^{f\,0.5}e^{-\left(M_2^S/C_y^f\right)} - q_{y,2}^{SE} - H_{y,2}^S$	Fraction of juvenile CoTS that transition to adults is dependent on availability of preferred coral [3].	
CoTS: age a = 3:5	$S_{y+1,a} = S_{y,a-1}e^{-\left(M_a^S/C_y^f\right)/(a-1)} - q_{y,a}^{SE} - H_{y,a}^S$	Adult CoTS mortality decreases with both age [4,5] and availability of fast-growing coral as their preferred prey [6].	1c
CoTS: age 6+	$S_{y+1,6+} = (S_{y,5} + S_{y,6+})e^{-\left(M_{6+}^S/C_y^f\right)} - q_{y,a}^{SE} - H_{y,6}^S$	CoTS mortality increases again at 6-years with senescent phase [7], while continuing to depend on coral availability.	1d
Terms for predation on CoTS			
Predation of juvenile CoTS by benthic invertebrates	$q_{y,1}^{SB} = \frac{p_1^{SB}S_{y,1}B_y}{S_{y,1} + p_1^{SB}B_y}$	Predation can be limited by juvenile CoTS abundance or handling time [8].	2a
Predation of CoTS by emperors	$q_{y,a}^{SE} = \frac{p_a^{SE}S_{y,a}E_y e^{-(\delta_{a,1}R_y)}}{S_{y,a}+p_a^{SE}E_y}$, $E_y = \frac{1}{15}\left(E_{y,1} + 2E_{y,2} + 3E_{y,3} + 4E_{y,4} + 5E_{y,5}\right)$, $\delta_{a,1} = \begin{cases}1; a = 1\\ 0; a \neq 1\end{cases}$	Predation increases with emperor age, but can be limited by CoTS abundance or handling time [9]. Rubble provides juvenile CoTS with a refuge from emperor predation [8,10].	2b
Coral population dynamics			
Coral groups	$C_{y+1}^g = C_y^g\left(1 + r_y^g - M_y^{g,Cyc} - M_y^{g,Ble} - q_y^{gS}\right) + \sum_{reefs}\alpha^C \mathrm{K}_y^C C_y^g\left(r_0^g/r_y^g\right)$ $(g = sa, ta, tt, mo, fa, po)$	Recruitment of coral is limited by competition [11] and declines with OA following growth rate [12].	3a
Structural coral cover	$C_y^f = C_y^{sa} + C_y^{ta} + C_y^{tt} + C_y^{mo}$		3b
Total coral cover	$C_y = C_y^{sa} + C_y^{ta} + C_y^{tt} + C_y^{mo} + C_y^{fa} + C_y^{po}$	Definition	3c
Coral diversity (evenness index)	$\mathcal{J} = -\frac{1}{ln(5)}\left(C_y^{sa}ln\left(C_y^{sa}\right) + C_y^{ta}ln\left(C_y^{ta}\right) + C_y^{mo}\,ln\left(C_y^{mo}\right) + C_y^{po}\,ln\left(C_y^{po}\right) + C_y^{fa}\,ln\left(C_y^{fa}\right)\right)$	Definition (excludes introduced thermally tolerant corals).	3d
Rubble cover	$R_{y+1} = R_y(1 - r^{consol}) + \sum_g C_y^g\left(M_y^{g,Cyc} + M_y^{g,Ble}\right)$	Rubble accumulation is driven by cyclone and bleaching events and is limited by consolidation.	3e
Terms for predation on coral			
Coral groups	$q_y^{gS} = p^{CS}S_y$ if $p^{CS}S_y < S_{pref}C_y^g$, $S_y = 0.2S_{y,1} + S_{y,2} + 2S_{y,3} + 4S_{y,4} + 8S_{y,5} + 8S_{y,6+}$ With predation applied sequentially for $(g = sa, ta, tt, mo, fa, po)$	Consumption of coral increases with the age class of CoTS [13]. CoTS consume faster-growing corals first [6].	4a

Description	Equation	Assumptions	#
Fish population dynamics			
Benthic invertebrates	$B_{y+1} = B_y\left(1 + r_0^B\left(1 - B_y/K_B\right) - q_y^{BT}\right)$	Recruitment is limited by carrying capacity. Limited evidence from other regions [14].	5a
Triggerfish (and cardinalfish)	$T_{y+1} = T_y\left(1 + r_0^T\left(1 - T_y/K_T\right) - q_y^{TG}\right)$	Recruitment is limited by territorial behaviour [15].	5b
Emperors<br>age *a* = 0	$E_{y+1,0} = \alpha_y^E\left(\left(E_{y,4} + 2E_{y,5}\right) + \sum_{reefs}\left(\dot{E}_{y,4} + 2\dot{E}_{y,5}\right)\right)$	Emperors breed from age 4-years with larval production doubling for each age-class.<br>Juveniles come from local reefs via seagrass nursery grounds (no larval retention).	5c
Emperors<br>age *a* = 1	$E_{y+1,1} = E_{y,0}$	Recruitment is modelled only for fish surviving at least to age 1.	5d
Emperors<br>age *a* = 2:5	$E_{y+1,a} = E_{y,a-1}\left(1 - \frac{M^E}{(a-1)(1+C_y)}E_{y,a-1}\right) - F_{y,a}^E$	Quadratic mortality term.<br>Mortality rate decreases with age and coral cover.	5e
Groupers<br>age *a* = 0	$G_{y+1,0} = \alpha^G\left(\lambda\left(G_{y,2} + 2S_{y,3} + 4G_{y,4} + 8S_{y,5}\right) + (1-\lambda)\sum_{reefs}\mathrm{K}_y^G\left(\dot{G}_{y,2} + 2\dot{G}_{y,3} + 4\dot{G}_{y,4} + 8\dot{G}_{y,5}\right)\right)$	Groupers breed from age 2-years with larval production doubling for each age-class.	5f
Groupers<br>age *a* = 1	$G_{y+1,1} = G_{y,0}$	Recruitment is modelled only for fish surviving at least to age 1.	5g
Groupers<br>age *a* = 2:5	$G_{y+1,a} = G_{y,a-1}\left(1 - \frac{M^G}{(a-1)}G_{y,a-1}\right) - F_{y,a}^G$	Quadratic mortality term.<br>Mortality rate decreases with age and coral cover.	5h
Terms for predation on fish			
Predation of benthic invertebrates by triggerfish	$q_y^{BT} = \frac{p^{BT}T_y}{B_y + p^{BT}T_y}e^{-(C_y+R_y)}$	Predation is reduced by coral and rubble cover [16].<br>Predation can be limited by benthic invertebrate abundance or handling time.	6a
Predation of triggerfish by groupers	$q_y^{TG} = \frac{p^{TG}G_y}{T_y+p^{TG}G_y}e^{-C_y}$, $G_y = \frac{1}{15}\left(G_{y,1} + 2G_{y,2} + 3G_{y,3} + 4G_{y,4} + 5G_{y,5}\right)$	Predation is reduced by coral cover.	6b

Supplementary Table 2: CoCoNet model equations for environmental effects on corals.

Description	Equation	Assumptions	No.
Terms for environmental effects on coral	$(\boldsymbol{g = sa, ta, tt, mo, fa, po})$		
Fraction of larvae retained on reefs	$\lambda = e^{-50/sites^{0.5}}$	Estimate based on high-resolution particle tracking experiments (Jim Greenwood, personal communication).	7a
Maximum cyclone induced mortality	$M_y^{g,Cyc} = \min\left(1.0, \left(0.28CAT_y - 0.24\right) r_0^g / r_y^g\right) \quad (g = sa, ta, tt)$<br>$M_y^{mo,Cyc} = 0.5\left(M_y^{sa,Cyc} + M_y^{fa,Cyc}\right) \quad (g = mo)$<br>$M_y^{g,Cyc} = \left(0.074CAT_y^2 - 0.24CAT_y + 0.24\right) r_0^g / r_y^g \quad (g = fa, po)$	Cyclone mortality is higher for fast-growing corals than slow-growing corals [17].<br>Cyclone mortality increases as growth rates fall with ocean acidification [18], approximately doubling by 2100 under SSP2-4.5.	7b
Maximum bleaching induced mortality	$M_y^{g,Ble} = 1 - e^{\left(-0.1e^{0.3max\left(0, DHW_y - S_y - \mathcal{T}_y^g\right)}\right)}$	Bleaching mortality is dependent on DHW exposure and the thermal tolerance of corals. Data from Sam Matthews suggest significantly lower mortality than shallow coral estimates [19]. Mortality at each reef is varied randomly $[0.5\ 1.0]M_y^{g,Ble}$.	7c
Intrinsic thermal tolerance	$\mathcal{T}_0^g = 3.5 - 5r_0^g$	Slower growing corals have higher thermal tolerance than faster growing corals [20].	7d
Thermal tolerance adaptation after bleaching	$\mathcal{T}_{y+1}^g = min\left(\mathcal{T}_y^g(1 + \mathcal{A})^{M_y^{g,Ble}}, \mathcal{T}_0^g + \mathcal{P}\right)$	The average thermal tolerance of corals surviving a bleaching increases within an upper bound [21].	7e
Influence of thermal adaptation and ocean acidification on growth	$r_{y+1}^g = \left(r_y^g\left(1 - 0.01\left(\mathcal{T}_y^g - \mathcal{T}_0^g\right)\right)\right)^{OA}$	Increased thermal tolerance is associated with lower coral growth rates.<br>Growth rates fall with increasing ocean acidification.	7f
Ocean acidification influence on cyclone damage and coral growth	$OA = 1 + k^g(1 - P^{OA})RCP^{0.5}$	Ocean acidification effects are larger on faster-growing corals [22] and increase with climate change.	7g

Supplementary Table 3: CoCoNet model equations for control of CoTS and fishing.

Description	Equation	Assumptions	No.
CoTS control			
Ecological threshold (for coral decline)	$S_{y,ecol} = 7.2$	Empirical estimate based on number of reefs controlled historically.	8a
Control dives (to reach ecological threshold)	$\mathcal{D}i_y = 4.18\left(\frac{S_{y,2}+S_{y,3}+S_{y,4}+S_{y,5}+S_{y,6+}}{\alpha}\right)^{0.67}$	Empirical relationship (analysis by Dan Gladish, CSIRO, personal communication).	8b
Fisheries catches			
Annual catch of emperors	$Catch_y^E = \frac{600000}{Rep_{<2004}^E}\, max\left(1.0\,,\ 2.5\left(1 - e^{-0.01e^{0.08(y-1940)}}\right)\right)$ $(y < 2004)$<br>$Catch_y^E = \frac{400000}{Rep_{\geq 2004}^E}$ $(y \geq 2004)$	Empirical fit to aggregated confidential reef-scale fisheries data.	8c
Annual catch of groupers	$Catch_y^G = \frac{1160000}{Rep_{<2004}^G}\, max\left(1.0\,,\ 2.5\left(1 - e^{-0.01e^{0.08(y-1940)}}\right)\right)$ $(y < 2004)$<br>$Catch_y^G = \frac{900000}{Rep_{\geq 2004}^G}$ $(y \geq 2004)$	Empirical fit to aggregated confidential reef-scale fisheries data.	8d

Supplementary Table 4: Definition of CoCoNet model variables.

Variable	Definition
Crown-of-thorns starfish	(age: a = 0, 1, 2, 3, 4, 5, 6+)
$\boldsymbol{S_{y,a}}$	Number of CoTS of age *a* at the start of year *y* ($\dot{S}_{y,a}$ for connected reefs)
$\boldsymbol{H^S_{y,a}}$	Fraction of CoTS of age *a* removed through control programs during year *y*
$\mathbf{K}^S_y$	Dispersal kernel elements for CoTS
Coral groups	(group: $g = sa, ta, mo, tt, fa, po$)
$\boldsymbol{C^g_y}$	Cover of coral group g at the start of year *y* ($\dot{C}^g_y$ for connected reefs)
$\boldsymbol{C_y}$	Total coral cover at the start of year *y*
$\boldsymbol{R_y}$	Coral rubble cover at the start of year *y*
$\boldsymbol{M^{g,Cyc}_y}$	Cyclone induced mortality of coral group g in year *y*
$\boldsymbol{M^{g,Ble}_y}$	Bleaching induced mortality of coral group g in year *y*
$\boldsymbol{\mathcal{T}^g_y}$	Thermal tolerance (in DHW) of coral group g in year *y*
$\mathbf{K}^C_y$	Dispersal kernel elements for corals
Fish groups	(age: a = 0, 1, 2, 3, 4, 5+)
$\boldsymbol{E_{y,a}}$	Number of emperors of age *a* at the start of year *y* ($\dot{E}_{y,a}$ for connected reefs)
$\boldsymbol{G_{y,a}}$	Number of groupers of age *a* at the start of year *y* ($\dot{G}_{y,a}$ for connected reefs)
$\boldsymbol{Catch^E_y}$	Catch of emperors in year *y* distributed across ages $a \geq 3$ (kg)
$\boldsymbol{Catch^G_y}$	Catch of groupers in year *y* distributed across ages $a \geq 3$ (kg)
$\mathbf{K}^G_y$	Dispersal kernel elements for groupers
Environmental conditions	
$\boldsymbol{CAT_y}$	Cyclone category within year *y* (1, 2, 3, 4, 5)
$\boldsymbol{DHW_y}$	Degree heating weeks at a reef over year *y*
Other interventions	
$\boldsymbol{\mathcal{S}_y}$	Effect of artificial shading or cooling (in DHW) at a reef over year *y*
$\boldsymbol{P^{OA}}$	Level of artificial protection from ocean acidification [0 1]

Supplementary Table 5: CoCoNet model parameter values.

Parameter	Description	Value	Estimation method	Reference
Crown-of-thorns starfish				
α^S	Maximum recruitment per CoTS (a = 2) on natal reefs	300000	Fitted to LTMP	[23]
M_a^S	Natural mortality of CoTS age a when preferred coral is abundant	0.8 (a =1,6)<br>0.0<br>(a =2-5)	Fitted to LTMP	-
p_1^{SB}	Juvenile CoTS consumed per benthic invertebrate per year	500	Pre-specified	Plausible estimate
p_1^{SE}	Juvenile CoTS consumed per emperor per year	500	Pre-specified	Plausible estimate
p_a^{SE}	Adult CoTS consumed per emperor per year ($a \geq 2$)	50	Pre-specified	Plausible estimate
ϵ	Conversion factor: control program CoTS ha^{-1} to CoTS per manta tow	0.015	Pre-specified	[24]
Coral groups				
r_0^{sa}	Intrinsic growth rate (including local recruitment) in year *y*	0.50 yr^{-1}	Pre-specified	[25]
r_0^{ta}	Intrinsic growth rate (including local recruitment) in year *y*	0.40 yr^{-1}	Pre-specified	[25]
r_0^{tt}	Intrinsic growth rate (including local recruitment) in year *y*	0.40 yr^{-1}	Pre-specified	[25]
r_0^{mo}	Intrinsic growth rate (including local recruitment) in year *y*	0.30 yr^{-1}	Pre-specified	[25]
r_0^{po}	Intrinsic growth rate (including local recruitment) in year *y*	0.15 yr^{-1}	Pre-specified	[25]
r_0^{fa}	Intrinsic growth rate (including local recruitment) in year *y*	0.10 yr^{-1}	Pre-specified	[25]
α^C	Maximum recruitment of coral group g from connected reefs	0.05	Fitted to LTMP	-
K_C	Coral carrying capacity	1.0	By definition	-
p^{CS}	Consumption rate of coral by COTS	0.0003	Empirical data	-
r^{consol}	Consolidation rate of coral rubble	0.17 yr^{-1}	Pre-specified	[26]
Fish groups				
K_B	Carrying capacity of benthic invertebrate CoTS predators	10000 ha^{-1}	Pre-specified	Guess
K_T	Carrying capacity of triggerfish	200 ha^{-1}	Pre-specified	[27]
α^B	Benthic invertebrate recruitment	0.5	Pre-specified	Plausible estimate
α^T	Triggerfish recruitment	0.8	Pre-specified	Plausible estimate
α^E	Emperor recruitment	0.0028	Fitted to LTMP	-
α^G	Grouper recruitment	4.0	Fitted to LTMP	-
M^G	Natural mortality of groupers	0.01	Pre-specified	[28,29]
M^E	Natural mortality of emperors	0.026	Pre-specified	[29]
p^{BT}	Maximum benthic invertebrates consumed per triggerfish per year	120	Pre-specified	Plausible estimate
p^{TG}	Maximum triggerfish consumed per grouper per year	70	Pre-specified	Plausible estimate

Parameter	Description	Value	Estimation method	Reference
Environmental effects on corals				
$\boldsymbol{RCP}$		Climate scenario	1.9 (SSP1-1.9)<br>2.6 (SSP1-2.6)<br>4.5 (SSP2-4.5)<br>7.0 (SSP3-7.0)<br>8.5 (SSP5-8.5)	Pre-specified [30]
$\mathcal{A}$	Adaptability of corals to thermal stress (if $\mathcal{A}$ = 1, thermal tolerance of corals doubles as mortality approaches 100%)	1	High if shuffling symbionts	Pre-specified
$\mathcal{P}$	Maximum thermal plasticity of corals (high uncertainty, some consensus that >10 DHW is highly unlikely on relevant timescales)	8 DHW	Loose consensus of geneticists	Pre-specified
$\boldsymbol{k^{sa}}$	Factor controlling decline in coral growth and recruitment from ocean acidification	0.0025	Pre-specified	[31,32]
$\boldsymbol{k^{ta}}$	Factor controlling decline in coral growth and recruitment from ocean acidification	0.0020	Pre-specified	[31,32]
$\boldsymbol{k^{mo}}$	Factor controlling decline in coral growth and recruitment from ocean acidification	0.0005	Pre-specified	[31,33]
$\boldsymbol{k^{po}}$	Factor controlling decline in coral growth and recruitment from ocean acidification	0.0010	Pre-specified	[31,34]
$\boldsymbol{k^{fa}}$	Factor controlling decline in coral growth and recruitment from ocean acidification	0.0020	Pre-specified	[31]
$\boldsymbol{k^{tt}}$	Factor controlling decline in coral growth and recruitment from ocean acidification	0.0010	Pre-specified	[31]

References

1 Wilmes, J. C., Schultz, D. J., Hoey, A. S., Messmer, V. & Pratchett, M. S. Habitat associations of settlement-stage crown-of-thorns starfish on Australia's Great Barrier Reef. *Coral Reefs* **39**, 1163-1174 (2020). https://doi.org/10.1007/s00338-020-01950-6

2 Pratchett, M. S. *et al.* Reproductive investment and fecundity of Pacific crown-of-thorns starfish (Acanthaster cf. solaris) on the Great Barrier Reef. *Mar Biol* **168** (2021). https://doi.org/10.1007/s00227-021-03897-w

3 Deaker, D. J. *et al.* The hidden army: corallivorous crown-of-thorns seastars can spend years as herbivorous juveniles. *Biol Letters* **16** (2020). https://doi.org/10.1098/rsbl.2019.0849

4 Keesing, J. K., Halford, A. R. & Hall, K. C. Mortality rates of small juvenile crown-of-thorns starfish Acanthaster planci on the Great Barrier Reef: implications for population size and larval settlement thresholds for outbreaks. *Mar Ecol Prog Ser* **597**, 179-190 (2018). https://doi.org/10.3354/meps12606

5 Rogers, J. G. D. & Plaganyi, E. E. Culling corallivores improves short-term coral recovery under bleaching scenarios. *Nat Commun* **13** (2022). https://doi.org/10.1038/s41467-022-30213-x

6 Pratchett, M. S. Changes in coral assemblages during an outbreak of Acanthaster planci at Lizard Island, northern Great Barrier Reef (1995-1999). *Coral Reefs* **29**, 717-725 (2010). https://doi.org/10.1007/s00338-010-0602-9

7 Pratchett, M. S., Caballes, C. F., Rivera-Posada, J. A. & Sweatman, H. P. A. Limits to Understanding and Managing Outbreaks of Crown-of-Thorns Starfish (Acanthaster Spp.). *Oceanography and Marine Biology: An Annual Review, Vol 52* **52**, 133-199 (2014). https://doi.org/Book_Doi 10.1201/B17143

8 Wilmes, J. C. *et al.* Contributions of pre- versus post-settlement processes to fluctuating abundance of crown-of-thorns starfishes (Acanthaster spp.). *Mar Pollut Bull* **135**, 332-345 (2018). https://doi.org/10.1016/j.marpolbul.2018.07.006

9 Dunic, J. C. & Baum, J. K. Size structuring and allometric scaling relationships in coral reef fishes. *J Anim Ecol* **86**, 577-589 (2017). https://doi.org/10.1111/1365-2656.12637

10 Keesing, J. K. Temporal patterns in the feeding and emergence behaviour of the crown-of-thorns starfish Acanthaster planci. *Mar Freshw Behav Phy* **25**, 209-232 (1995). https://doi.org/Doi/10.1080/10236249509378919

11 Evensen, N. R., Bozec, Y. M., Edmunds, P. J. & Mumby, P. J. Scaling the effects of ocean acidification on coral growth and coral-coral competition on coral community recovery. *Peerj* **9** (2021). https://doi.org/10.7717/peerj.11608

12 Smith, J. N. *et al.* Shifts in coralline algae, macroalgae, and coral juveniles in the Great Barrier Reef associated with present-day ocean acidification. *Global Change Biol* **26**, 2149-2160 (2020). https://doi.org/10.1111/gcb.14985

13 Keesing, J. K. & Lucas, J. S. Field Measurement of Feeding and Movement Rates of the Crown-of-Thorns Starfish Acanthaster-Planci (L). *J Exp Mar Biol Ecol* **156**, 89-104 (1992). https://doi.org/Doi 10.1016/0022-0981(92)90018-6

14 Rodriguez-Troncoso, A. P., Rodriguez-Zaragoza, F. A., Mayfield, A. B. & Cupul-Magana, A. L. Temporal variation in invertebrate recruitment on an Eastern Pacific coral reef. *J Sea Res* **145**, 8-15 (2019). https://doi.org/10.1016/j.seares.2018.12.007

15 Bean, K., Jones, G. P. & Caley, M. J. Relationships among distribution, abundance and microhabitat specialisation in a guild of coral reef triggerfish (family Balistidae). *Mar Ecol Prog Ser* **233**, 263-272 (2002). https://doi.org/10.3354/meps233263

16 Fabricius, K. E., De'ath, G., Noonan, S. & Uthicke, S. Ecological effects of ocean acidification and habitat complexity on reef-associated macroinvertebrate communities. *P Roy Soc B-Biol Sci* **281** (2014). https://doi.org/DOI 10.1098/rspb.2013.2479

17 Condie, S. A., Plaganyi, E. E., Morello, E. B., Hock, K. & Beeden, R. Great Barrier Reef recovery through multiple interventions. *Conserv Biol* **32**, 1356-1367 (2018). https://doi.org/10.1111/cobi.13161

18 Madin, J. S., Hughes, T. P. & Connolly, S. R. Calcification, Storm Damage and Population Resilience of Tabular Corals under Climate Change. *Plos One* **7** (2012). https://doi.org/10.1371/journal.pone.0046637

19 Hughes, T. P. *et al.* Global warming and recurrent mass bleaching of corals. *Nature* **543**, 373-+ (2017). https://doi.org/10.1038/nature21707

20 Muir, P. R., Marshall, P. A., Abdulla, A. & Aguirre, J. D. Species identity and depth predict bleaching severity in reef-building corals: shall the deep inherit the reef? *Proc Biol Sci* **284** (2017). https://doi.org/10.1098/rspb.2017.1551

21 Matz, M. V., Treml, E. A., Aglyamova, G. V. & Bay, L. K. Potential and limits for rapid genetic adaptation to warming in a Great Barrier Reef coral. *Plos Genet* **14** (2018). https://doi.org/10.1371/journal.pgen.1007220

22 Comeau, S., Edmunds, P. J., Spindel, N. B. & Carpenter, R. C. Fast coral reef calcifiers are more sensitive to ocean acidification in short-term laboratory incubations. *Limnol Oceanogr* **59**, 1081-1091 (2014). https://doi.org/10.4319/lo.2014.59.3.1081

23 Caballes, C. F., Pratchett, M. S., Raymundo, M. L. & Rivera-Posada, J. A. Environmental Tipping Points for Sperm Motility, Fertilization, and Embryonic Development in the Crown-of-Thorns Starfish. *Diversity-Basel* **9** (2017). https://doi.org/10.3390/d9010010

24 Moran, P. J. & Death, G. Estimates of the Abundance of the Crown-of-Thorns Starfish Acanthaster-Planci in Outbreaking and Non-Outbreaking Populations on Reefs within the Great-Barrier-Reef. *Mar Biol* **113**, 509-515 (1992). https://doi.org/Doi 10.1007/Bf00349178

25 Hughes, T. P. *et al.* Global warming transforms coral reef assemblages. *Nature* **556**, 492-+ (2018). https://doi.org/10.1038/s41586-018-0041-2

26 Biggs, B. C. Harnessing Natural Recovery Processes to Improve Restoration Outcomes: An Experimental Assessment of Sponge-Mediated Coral Reef Restoration. *Plos One* **8** (2013). https://doi.org/10.1371/journal.pone.0064945

27 Kavanagh, K. D. & Olney, J. E. Ecological correlates of population density and behavior in the circumtropical black triggerfish Melichthys niger (Balistidae). *Environ Biol Fish* **76**, 387-398 (2006). https://doi.org/10.1007/s10641-006-9044-1

28 Mapstone, B. D. *et al.* Management strategy evaluation for line fishing in the Great Barrier Reef: Balancing conservation and multi-sector fishery objectives. *Fish Res* **94**, 315-329 (2008). https://doi.org/10.1016/j.fishres.2008.07.013

29 Little, L. R. *et al.* Modelling multi-species targeting of fishing effort in the Queensland Coral Reef Fin Fish Fishery. (James Cook University, Townsville, 2008).

30 McWhorter, J. K. *et al.* The importance of 1.5 degrees C warming for the Great Barrier Reef. *Global Change Biol* **28**, 1332-1341 (2022). https://doi.org/10.1111/gcb.15994

31 Dove, S. G. *et al.* Future reef decalcification under a business-as-usual CO2 emission scenario. *P Natl Acad Sci USA* **110**, 15342-15347 (2013). https://doi.org/10.1073/pnas.1302701110

32 Albright, R., Mason, B., Miller, M. & Langdon, C. Ocean acidification compromises recruitment success of the threatened Caribbean coral Acropora palmata. *P Natl Acad Sci USA* **107**, 20400-20404 (2010). https://doi.org/10.1073/pnas.1007273107

33 Browne, N. K. Spatial and temporal variations in coral growth on an inshore turbid reef subjected to multiple disturbances. *Mar Environ Res* **77**, 71-83 (2012). https://doi.org/10.1016/j.marenvres.2012.02.005

34 Fabricius, K. E., Noonan, S. H. C., Abrego, D., Harrington, L. & De'ath, G. Low recruitment due to altered settlement substrata as primary constraint for coral communities under ocean acidification. *P Roy Soc B-Biol Sci* **284** (2017). https://doi.org/10.1098/rspb.2017.1536
