# Supplementary material for: Protection of coral reef fish delivers ecosystem-critical biocontrol of coral-eating starfish across the Great Barrier Reef
Source: Nat Ecol Evol. 2025 Nov 28;10(1):117–27. doi: 10.1038/s41559-025-02916-z (PMC12789020; doi:10.1038/s41559-025-02916-z)
Supplement: Supplementary file 2 — Reporting Summary [file 41559_2025_2916_MOESM2_ESM.pdf]

nature portfolio

Corresponding author(s): Scott Condie

Last updated by author(s): Oct 27, 2025

Reporting Summary

Nature Portfolio wishes to improve the reproducibility of the work that we publish. This form provides structure for consistency and transparency in reporting. For further information on Nature Portfolio policies, see our Editorial Policies and the Editorial Policy Checklist.

Statistics

For all statistical analyses, confirm that the following items are present in the figure legend, table legend, main text, or Methods section.

n/a	Confirmed	
☐	☒	The exact sample size (*n*) for each experimental group/condition, given as a discrete number and unit of measurement
☐	☒	A statement on whether measurements were taken from distinct samples or whether the same sample was measured repeatedly
☐	☒	The statistical test(s) used AND whether they are one- or two-sided<br>*Only common tests should be described solely by name; describe more complex techniques in the Methods section.*
☐	☒	A description of all covariates tested
☒	☐	A description of any assumptions or corrections, such as tests of normality and adjustment for multiple comparisons
☐	☒	A full description of the statistical parameters including central tendency (e.g. means) or other basic estimates (e.g. regression coefficient) AND variation (e.g. standard deviation) or associated estimates of uncertainty (e.g. confidence intervals)
☒	☐	For null hypothesis testing, the test statistic (e.g. *F*, *t*, *r*) with confidence intervals, effect sizes, degrees of freedom and *P* value noted<br>*Give P values as exact values whenever suitable.*
☒	☐	For Bayesian analysis, information on the choice of priors and Markov chain Monte Carlo settings
☐	☒	For hierarchical and complex designs, identification of the appropriate level for tests and full reporting of outcomes
☒	☐	Estimates of effect sizes (e.g. Cohen's *d*, Pearson's *r*), indicating how they were calculated

Our web collection on statistics for biologists contains articles on many of the points above.

Software and code

Policy information about availability of computer code

Data collection	All study data was generated by the CoCoNet model developed within the NetLogo modelling environment (version 6.4.0) The CoCoNet model has been described in the referenced published literature. The code used in the study can be accessed at http://datadryad.org/share/xxGUGVuuflXrdIysXp3TJyM5BajOJYuKmItQt2AGYe8
Data analysis	No custom algorithms - analysis limited to standard time series and percentiles as available in all statistical packages.

For manuscripts utilizing custom algorithms or software that are central to the research but not yet described in published literature, software must be made available to editors and reviewers. We strongly encourage code deposition in a community repository (e.g. GitHub). See the Nature Portfolio guidelines for submitting code & software for further information.

Data

Policy information about availability of data

All manuscripts must include a data availability statement. This statement should provide the following information, where applicable:

- Accession codes, unique identifiers, or web links for publicly available datasets
- A description of any restrictions on data availability
- For clinical datasets or third party data, please ensure that the statement adheres to our policy

The model data generated in this study can all be accessed at http://datadryad.org/share/xxGUGVuuflXrdIysXp3TJyM5BajOJYuKmItQt2AGYe8 (also provided with the submission).

Research involving human participants, their data, or biological material

Policy information about studies with human participants or human data. See also policy information about sex, gender (identity/presentation), and sexual orientation and race, ethnicity and racism.

Reporting on sex and gender	No human participants, human data or biological materials were used in the study.
Reporting on race, ethnicity, or other socially relevant groupings	No human participants, human data or biological materials were used in the study.
Population characteristics	No human participants, human data or biological materials were used in the study.
Recruitment	No human participants, human data or biological materials were used in the study.
Ethics oversight	No human participants, human data or biological materials were used in the study.

Note that full information on the approval of the study protocol must also be provided in the manuscript.

Field-specific reporting

Please select the one below that is the best fit for your research. If you are not sure, read the appropriate sections before making your selection.

☐ Life sciences ☐ Behavioural & social sciences ☒ Ecological, evolutionary & environmental sciences

For a reference copy of the document with all sections, see nature.com/documents/nr-reporting-summary-flat.pdf

Ecological, evolutionary & environmental sciences study design

All studies must disclose on these points even when the disclosure is negative.

Study description	Each model experiment represented a single intervention scenario and was run as an ensemble of 20 replicates differing only in the stochastic environmental conditions they were exposed to (e.g. ocean circulation, tropical cyclones, marine heat waves). Results from 17 scenarios were reported.
Research sample	The model tracked annual populations of 5 coral functional groups, crown-of-thorns starfish (age-structured), a benthic invertebrate group, and 3 fish groups (age-structured).
Sampling strategy	Model outputs were saved at every time-step (annual).
Data collection	Model outputs were saved at every time-step (annual).
Timing and spatial scale	All model runs covered the entire Great Barrier Reef region (latitudes 10-25 S) and the period 1956-2100. Annual outputs were saved for the period 1986-2100.
Data exclusions	No data were excluded.
Reproducibility	Model runs were repeated to ensure that results were identical (identical random-seeds were used in the modelling).
Randomization	Model runs used identical series of random-seeds, so that differences between scenarios were entirely due to differences in the interventions being evaluated.
Blinding	Blinding was not relevant as model setting were controlled by random number generation inside the model.

Did the study involve field work? ☐ Yes ☒ No

Reporting for specific materials, systems and methods

We require information from authors about some types of materials, experimental systems and methods used in many studies. Here, indicate whether each material, system or method listed is relevant to your study. If you are not sure if a list item applies to your research, read the appropriate section before selecting a response.

Materials & experimental systems

n/a	Involved in the study	
☒	☐	Antibodies
☒	☐	Eukaryotic cell lines
☒	☐	Palaeontology and archaeology
☒	☐	Animals and other organisms
☒	☐	Clinical data
☒	☐	Dual use research of concern
☒	☐	Plants

Methods

n/a	Involved in the study	
☒	☐	ChIP-seq
☒	☐	Flow cytometry
☒	☐	MRI-based neuroimaging

Plants

Seed stocks	No plant material was used in the study.
Novel plant genotypes	No plant material was used in the study.
Authentication	No plant material was used in the study.
